# Insulin and Insulin-Sensitizing Drugs in Neurodegeneration: Mitochondria as Therapeutic Targets

**DOI:** 10.3390/ph2030250

**Published:** 2009-12-23

**Authors:** Susana Cardoso, Renato Santos, Sonia Correia, Cristina Carvalho, Xiongwei Zhu, Hyoung-Gon Lee, Gemma Casadesus, Mark A. Smith, George Perry, Paula I. Moreira

**Affiliations:** 1 Center for Neuroscience and Cell Biology, University of Coimbra, 3000-354 Coimbra, Portugal; 2 Department of Life Sciences, Faculty of Sciences and Technology, University of Coimbra, 3000-354 Coimbra, Portugal; 3 Institute of Physiology, Faculty of Medicine, University of Coimbra, 3000-354 Coimbra, Portugal; 4 School of Medicine, Case Western Reserve University, Cleveland, OH 44106, USA; 5 College of Sciences, The University of Texas at San Antonio, TX 78249, USA

**Keywords:** Alzheimer’s disease, amyotrophic lateral sclerosis, Huntington’s disease, insulin, mitochondria, neurodegeneration, Parkinson’s disease, PPARs agonists

## Abstract

Insulin, besides its glucose lowering effects, is involved in the modulation of lifespan, aging and memory and learning processes. As the population ages, neurodegenerative disorders become epidemic and a connection between insulin signaling dysregulation, cognitive decline and dementia has been established. Mitochondria are intracellular organelles that despite playing a critical role in cellular metabolism are also one of the major sources of reactive oxygen species. Mitochondrial dysfunction, oxidative stress and neuroinflammation, hallmarks of neurodegeneration, can result from impaired insulin signaling. Insulin-sensitizing drugs such as the thiazolidinediones are a new class of synthetic compounds that potentiate insulin action in the target tissues and act as specific agonists of the peroxisome proliferator-activated receptor gamma (PPAR-γ). Recently, several PPAR agonists have been proposed as novel and possible therapeutic agents for neurodegenerative disorders. Indeed, the literature shows that these agents are able to protect against mitochondrial dysfunction, oxidative damage, inflammation and apoptosis. This review discusses the role of mitochondria and insulin signaling in normal brain function and in neurodegeneration. Furthermore, the potential protective role of insulin and insulin sensitizers in Alzheimer´s, Parkinson´s and Huntington´s diseases and amyotrophic lateral sclerosis will be also discussed.

## 1. Introduction

Insulin is a peptide hormone composed of 51 aminoacids and has a molecular weight of about 6,000 Da. It is synthesized in the pancreatic β-cells that when stimulated release the hormone by exocytosis into islet capillary blood [1]. Insulin will then bind to its receptor (IR) leading to glucose uptake, muscle and adipocytes metabolism and inhibition of gluconeogenesis in the liver [2]. Situations that impair any of the above mentioned events will ultimately lead to an impaired glucose uptake defined as diabetes mellitus. Although diabetes was considered a peripheral disease, it is becoming widely accepted that diabetes also affects the central nervous system (CNS) [1]. Insulin signaling is crucial for growth and survival [3] and despite studies in lower metazoans showing that reduced insulin signaling extends life span [4,5], in mammals things are not so linear because insulin/IR exert opposite effects whether they are located in the CNS or the periphery [6]. Indeed, the loss of IR in adipose tissue promotes longevity, whereas its loss in the hepatic tissues causes diabetes [7]. Perhaps in conditions of systemic insulin/insulin growth factor 1 (IGF-1) signaling reduction the metabolic syndromes (such as diabetes) that stem from the liver hide the potential health benefits of reduced insulin/IGF-1 signaling in other tissues, such as the brain. Since the identification of insulin and IR in the brain, insulin function in the CNS has been under intense debate. Epidemiological and clinical studies suggest a connection between diabetes, high insulin levels and cognitive impairment [1]. Recent studies show that insulin/IR are involved in brain functions such as learning and memory [8,9], whereas their impairment has been linked to the development of age-related neurodegenerative disorders [10,11,12]. Aging is a universal process and the major risk factor for several neurodegenerative disorders including Parkinson´s (PD), Huntington´s (HD) and Alzheimer´s (AD) diseases and amyotrophic lateral sclerosis (ALS). In the brain, as well as in other organs, aging is associated with mitochondria impairment, increased oxidative damage [13], hyperinsulinemia and impaired insulin sensitivity [14,15]. 

In eukaryotic cells, mitochondria are the main site of energy production, where ATP is produced via oxidative metabolism [16]. ATP production by mitochondria involves two major steps: (1) the oxidation of reducing equivalents that are produced by enzymatic pathways involved in the metabolism of glucose, fatty acids and other substrates and (2) the phosphorylation of ADP to ATP (*i.e.*, oxidative phosphorylation) [16]. The alteration of mitochondrial energy metabolism leads to reduced ATP production, impaired calcium buffering, and generation of reactive oxygen species (ROS). The generation of ROS is increasingly recognized as playing an important role in both aging and neurodegenerative disorders where mitochondria are both sources and targets of these reactive species [15,17,18]. 

Peroxisome proliferator-activated receptors (PPARs) are ligand-activated transcription factors that belong to the nuclear hormone receptor superfamily [19]. Nuclear receptors bind directly to DNA regulating gene expression through transcriptional co-activation [20]. Initially, it was thought that PPARs activity was limited to lipid metabolism and glucose homeostasis. However, subsequent studies revealed that PPARs are also involved in several biological functions, such as cell proliferation, differentiation and apoptosis [21]. PPAR-γ is the best characterized isoform mainly because it regulates serum glucose levels and insulin sensitivity, therefore being widely used in the treatment of diabetes [22,23]. Since PPAR-γ is also expressed in neurons and astrocytes raised the hypothesis that PPAR-γ could be a potential therapeutic target in CNS disorders [20]. Several studies demonstrated that PPAR-γ agonists improve disease-related symptomology and pathology in several animal models [24] by directly improving mitochondrial function and, ultimately, ATP production [25,26]. 

In this review we will discuss the role of mitochondria and insulin signaling in brain physiologic and pathologic conditions. The potential protective role of insulin and insulin-sensitizing agents in AD, HD and PD and ALS will be also discussed.

## 2. Mitochondria and the Brain

Mitochondria are essential organelles for mammalian cell survival since they are the main producers of ATP, an energy molecule crucial for cells functioning. Mitochondria, unlike all the other organelles, have their own DNA that encodes components of the oxidative phosphorylation system (OXPHOS) [27,28]. However, mitochondria remain dependent on the nucleus for the production of several subunits of the respiratory chain complexes and proteins related to transcription, translation, replication and repair. The OXPHOS is located in the inner mitochondrial membrane and is composed by five respiratory chain complexes, NADH-ubiquinone oxidoreductase (Complex I), succinate-ubiquinone oxidoreductase (Complex II), ubiquinone-cytochrome c reductase (Complex III), cytochrome c oxidase (Complex IV) and ATP synthase (Complex V). There are two electron carriers, ubiquinone (coenzyme Q), located in the inner mitochondrial membrane and cytochrome c, located in the intermembrane space [29]. Reducing equivalents produced in the Krebs cycle and in the β-oxidation pass through complexes I to IV and the energy generated by the electron transfer is used to pump protons from the mitochondrial matrix into the intermembrane space creating an electrochemical proton gradient used to drive complex V to generate ATP [30]. However, this system is not perfect and a small proportion of the electrons flowing through complexes I and III react with oxygen forming superoxide anion that can be converted into other ROS [27]. 

ROS have a dual role in cells, acting as both beneficial or harmful species [31]. In response to certain stimuli cells produce low/moderate levels of ROS that have physiological functions intervening in several cellular signaling pathways, therefore acting as second messengers [31,32]. Taking into account that ROS actions are cell-context dependent, low/moderate levels of ROS have the ability to activate (1) IR, mimicking insulin effects in the modulation of metabolism and cell growth [33], (2) Akt, inducing the phosphorylation of Hsp27 [34] and (3) mitochondrial enzyme activities [35]. Also, treatment of cells with H_2_O_2_ leads to the activation of transcription factors such as activator protein-1 (AP-1) and nuclear factor κB (NF-κB) [36]. In contrast, excessive ROS formation will lead to damage of proteins, lipids and nucleic acids. Moreover, situations of increased oxidative stress and mitochondrial calcium overload promote the opening of the permeability transition pore (PTP), a situation in which the mitochondrial proton motive force is disrupted. PTP opening will lead to the release of pro-apoptotic proteins like cytochrome c, which induce the caspase-mediated apoptosis [31]. In order to overcome the oxidative insult, cells possess a variety of enzymatic and non-enzymatic antioxidant defenses. However, if an imbalance between antioxidant defenses and ROS formation occurs, oxidative damage of cells will happen contributing to the development of neurodegenerative diseases [37]. 

In 1956 Harman proposed the free radical theory of aging that postulates that free radicals play a central role in the aging process [38]. The brain is extremely sensitive to oxidative damage due to its high oxygen demand, its high content of oxidisable polyunsaturated fatty acids, the presence of redox-active metals [18,39] and a low activity of antioxidant enzymes [18,30]. Since oxidative stress increases with age and mitochondria are both targets and sources of ROS, there is the assumption that mitochondria have a central role in aging and neurodegenerative disorders [40]. The ROS generated by the OXPHOS induce mutations in the mtDNA potentiating OXPHOS impairment. Consequently, the impaired OXPHOS potentiates ROS production increasing the number of mtDNA mutations [27]. Although the majority of the literature supports the free radical (mitochondrial) theory of aging, there are some studies that do not confirm this hypothesis. Studies performed with murine embryonic fibroblasts from the “mutator mice” that accumulate mtDNA mutations in an age-dependent way show that cells and tissues from adult mice did not exhibit increased ROS production neither oxidative damage [41] suggesting that oxidative stress is not involved in age-associated mtDNA mutations [27]. This is supported by a previous study showing that double-strand breaks in the mtDNA could contribute to mtDNA mutations during aging [42]. Others studies using C. elegans, a model often used to evaluate the effects of mitochondrial function on longevity [43], demonstrated that mutations in complex III of the mitochondrial electron transport chain (ETC) [44] leads to low oxygen consumption, decreased sensitivity to ROS and increased life span. Additionally, it was shown that lowering the activity of OXPHOS with RNA interference during development extended adult life-span [45]. However, and in contrast to insulin/IGF-1 signaling that affects longevity during adulthood, the decrease in ETC only extends lifespan when occurring during larval development [7]. Despite the fact that neurodegenerative disorders have disparate clinical features, they are characterized by mitochondrial dysfunction and oxidative stress [29]. 

## 3. Mitochondria and Neurodegeneration

AD is a progressive age-dependent neurodegenerative disorder and the most common form of dementia, accounting for 50–70% of dementia cases. While less than 5% of AD cases are familial [46] and associated with mutations in amyloid β protein precursor (APP) and presenilins 1 and 2 (PS1 and PS2), the majority of AD cases are sporadic in origin and involve genetic and environmental factors that taken alone are not sufficient to develop the disease [47]. AD is characterized by progressive cognitive decline and the presence of Aβ plaques and tau neurofibrillary tangles [15,48]. APP can be processed by two pathways, amyloidogenic and non-amyloidogenic, Aβ being generated by the abnormal processing of APP through the amyloidogenic pathway [49]. AD is associated with mitochondrial abnormalities, oxidative damage, inflammation and the loss of synaptic function, synapses and neurons [49]. 

**Figure 1:**
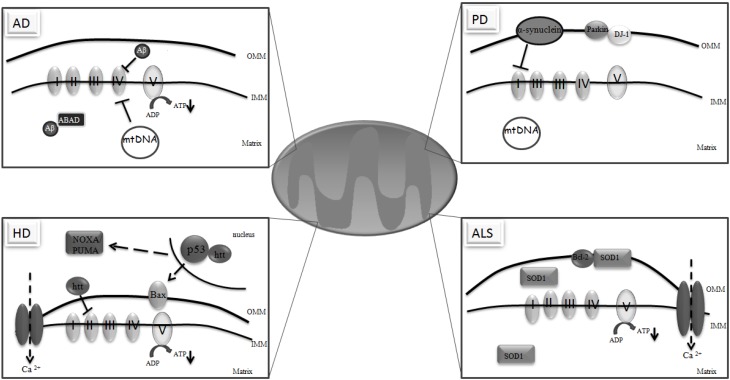
Mitochondrial dysfunction in neurodegeneration. In Alzheimer´s disease (AD), Aβ accumulates in mitochondria and binds to Aβ-binding alcohol dehydrogenase (ABAD) inhibiting complex IV, potentiating reactive oxygen species (ROS) formation and decreasing ATP production. AD pathology can also be influenced by mutations in the mtDNA, since mtDNA from AD subjects have a higher rate of mutations. In Parkinson´s Disease (PD), complex I activity is impaired contributing to the formation of high levels of ROS. Many of the genes involved in PD are also associated with mitochondrial dysfunction. α-synuclein overexpression potentiates mitochondrial impairment and oxidative stress. Parkin associates with the outer mitochondrial membrane (OMM) protecting mitochondria against ROS release and caspase activation. DJ-1 is an integral protein that participates in the oxidative stress response and protects against the loss of dopaminergic neurons. In PD, the level of mtDNA mutations is also associated with respiratory chain deficiencies. In Huntington’s disease (HD), mutant huntingtin (htt) compromises complex II activity, ATP production and the calcium (Ca^2+^) buffering capacity. htt also affects mitochondrial function through its interaction with p53 in the nucleus leading to upregulation of BAX and PUMA, two pro-apoptotic proteins. In amyotrophic lateral sclerosis (ALS), mutant Cu/Zn superoxide dismutase (SOD1) that is localized in the outer mitochondrial membrane (OMM), intermembrane space (IMM) and mitochondrial matrix, impairs mitochondrial respiration and ATP synthesis as well as the mitochondrial Ca^2+^ loading capacity. Mutant SOD1 binds to Bcl-2 on the OMM blocking its anti-apoptotic activity.

Accumulating evidence suggests mitochondria are important players in the mechanism by which Aβ triggers synaptic failure and neurodegeneration [15,18,50,51,52]. *In vivo* studies show accumulation of Aβ in brain mitochondria of AD patients [53]. Further, *in vitro* studies show that NT2 neuronal cells without mtDNA are not killed by Aβ [54]. Data from our laboratory show that Aβ induces mitochondrial dysfunction by potentiating respiratory chain impairment, uncoupling of the OXPHOS, decreasing ATP levels and increasing the susceptibility to PTP opening and H_2_O_2_ production [55,56,57]. Also, Lustbader and colleagues [58] demonstrated that Aβ binds to the mitochondrial-matrix protein Aβ-binding alcohol dehydrogenase (ABAD) (Figure 1) and the blockage of this interaction suppresses Aβ-induced apoptosis and free radical generation in neurons. These results suggest that mitochondria are key players in the toxicity induced by Aβ. It has been also shown that oxidative damage occurs before Aβ deposition [59,60] and that the upregulation of genes related to mitochondrial metabolism and apoptosis occurs even earlier and co-localizes with the neurons undergoing oxidative damage [61].

Tau protein is involved in the stabilization of microtubules, which is important in the generation and maintenance of neurites. In AD, tau accumulation in neurons inhibits APP transport into axons and dendrites leading to neuronal degeneration [62]. Transgenic mice overexpressing the P301L mutant human tau revealed impaired mitochondrial respiration, modified lipid peroxidation levels and up-regulation of antioxidant enzymes [63]. However, the mechanisms underlying these effects remain unknown.

Positron emission tomography (PET) studies revealed that AD is associated with brain metabolism impairment, which precedes neuropsychological impairment and atrophy [64,65]. It was observed that postmortem brain and fibroblasts from AD patients have an impairment of the three key TCA cycle enzymes, pyruvate dehydrogenase, isocitrate dehydrogenase and α-ketoglutarate dehydrogenase [66,67,68,69,70]. Furthermore, it has been demonstrated that Aβ inhibits cytochrome c oxidase (COX) [71] (Figure 1) therefore increasing free-radical generation [72]. Deficient COX activity has been found in different brain regions [73,74], platelets [75] and fibroblasts [76] from sporadic AD patients, occurring at all stages of the disease, including mild cognitive impairment (MCI) [77].

King and Attardi [78] developed a cybrid model, lacking their own mtDNA, in which exogenous mtDNA from AD and control patients were introduced. In this way they found that the phenotypic differences observed were due to donor mtDNA amplification and not from nuclear or environmental factors [78]. Later on, it was demonstrated that sporadic AD cybrids present reduced COX activity, a decrease in ATP levels and increased oxidative stress [79] and develop populations of abnormal and damaged mitochondria due to increased AD mtDNA replication [80] (Figure 1). In addition, it was reported that AD cybrids manifest a decrease in mitochondrial membrane potential, increased cytochrome c release and caspase-3 activity when compared to control cybrids [81]. Furthermore, those effects were enhanced when exposing AD cybrids to Aβ1-40, suggesting a role for mtDNA in mitochondrial dysfunction in AD degeneration [81]. A previous study made in AD, aged and younger control subjects demonstrated that the brains of AD and elderly subjects had a lower COX activity and a higher aggregate burden of mutations in mtDNA when compared to younger individuals [82]. AD has also been linked to mitochondria due to data from epidemiologic, neuropsychological, biomarker, and cell studies suggesting that mitochondrial inheritance could also influence AD risk and pathology [83]. For instance, evidence suggest that the European mtDNA haplogroups J and UK protect against AD and PD, and are also associated with increased longevity [84]. However, studies show that whenever AD patients have a demented parent, it is often the mother [85]. More recently, the Framingham Offspring Study demonstrated that non-demented and middle aged individuals whose mother suffer from AD have worse neuropsychological test performance than those with an AD-affected father or no affected parent [86]. Moreover, it was also reported that cybrid cell lines containing mtDNA from individuals with mothers suffering from AD possess lower COX activity than those containing mtDNA from subjects with fathers suffering from this disease [87]. Despite controversial data showing that pathogenic inherited mtDNA do not constitute a major ethological factor in sporadic AD [88], the majority of studies support the notion that inheritance could influence mitochondrial function and thereby AD risk and pathology.

De la Monte and Wands [89] examined postmortem brain tissue from AD patients with different degrees of severity and found that the severity of AD was related to impairments in mitochondrial gene expression, namely in complex IV of the mitochondrial respiratory chain, increased levels of p53 and molecular indexes of oxidative stress, such as up-regulation of nitric oxide synthase (NOS) and NADPH-oxidase (NOX). However, no differences in the levels of mitochondrial complexes I and II mRNA expression were found, suggesting that these components are preserved in AD, even in advanced stages of the disease [89]. Increased ROS production could therefore lead to mitochondrial dysfunction blocking electron transport, thus decreasing oxygen consumption and ATP generation.

Previous studies from our laboratory also showed altered levels of mtDNA and COX in neurons prior to the formation of neurofibrillary tangles [90], which suggest that mitochondrial abnormalities are the earliest cytopathological changes in AD. We also observed an increase in mtDNA and COX in the cytoplasm and in vacuoles associated with lipofuscin, considered the site of mitochondrial degradation by autophagy [90]. Subsequent studies also demonstrated an increased localization of COX and lipoic acid, a sulfur-containing cofactor necessary for the activity of some mitochondrial enzyme complexes, in autophagic vacuoles and lipofuscin in the brain of AD cases suggesting altered autophagic degradation of mitochondria [91,92]. The increase in mitochondrial degradation products that occur in AD vulnerable neurons could be due to an increase in mitochondria turnover by autophagy or a reduction of proteolytic turnover leading to mtDNA and mitochondrial protein accumulation. Despite these evidences there is still some controversy about COX involvement in the induction of oxidative stress in AD. Fukui and colleagues [93] using a COXd/AD mice demonstrated that COX deficiency in neurons results in decreased Aβ accumulation and reduced oxidative stress in CNS suggesting that COX impairment and oxidative damage in AD could be two independent consequences of Aβ intra- and extracellular accumulation.

PD is the second most common neurodegenerative disorder that begins by causing motor dysfunction but ultimately affects the mind and personality [20]. This disease is clinically characterized by progressive rigidity, bradykinesia and tremor and pathologically by the degeneration of pigmented neurons in the substantia nigra and by the presence of intraneuronal proteinaceous cytoplasmic inclusions that immunostain for α-synuclein and ubiquitin, designated Lewy Bodies [20,40]. The involvement of mitochondrial dysfunction in PD arose from the finding that 1-methyl-4-phenyl-1,2,3,6-tetrahydropyridine (MPTP), a synthetic opiate, caused Parkinsonism in drug addicted individuals [87]. MPTP is metabolized to MPP^+^ in glial cells and this metabolite inhibits the complex I of the mitochondrial respiratory chain [94]. In addition to MPTP also rotenone, another complex I inhibitor, originate a parkinsonian phenotype characterized by oxidative damage and nigral degeneration [95,96]. These results support the involvement of mitochondrial dysfunction in PD. Similarly, cybrids containing mtDNA from PD patients show reduced complex I activity and an increased susceptibility to MPP^+^ [97,98]. In addition to the defect in mitochondrial complex I, many of the genes involved in PD, such as α-synuclein, parkin, DJ-1, PINK-1 [99,100,101], LRRK-2 [102,103] and HTRA2 [104,105] have also a direct or indirect effect in mitochondria function. For instance, although there is no direct link of α-synuclein to mitochondria, the addition of MPTP to α-synuclein overexpressing mice leads to the formation of large and grossly deformed mitochondria, increase in oxidative stress and enhancement of nigral pathology [106]. Similarly, Hsu and colleagues [107] reported that the overexpression of α-synuclein impairs mitochondrial function and leads to increased oxidative damage. More recently, Devi and co-workers [108] demonstrated that α-synuclein accumulates in the mitochondria of striatum and substantia nigra of PD patients inducing oxidative stress and impairment of complex I activity (Figure 1). In contrast, α-synuclein null mice are resistant to MPTP and malonate actions, thereby implicating mitochondria in α-synuclein mediated toxicity [109,110]. Parkin, a protein involved in the degradation of oxidatively damaged proteins, associates with the outer mitochondrial membrane protecting mitochondria against swelling and ROS release [17] and caspase activation [111] whereas parkin-deficient mice present mitochondrial dysfunction and oxidative damage [112]. Parkin was also found in mitochondria from proliferating cells associated with mitochondrial transcription factor A (Tfam) therefore enhancing mitochondrial biogenesis [113]. Nevertheless, mitochondrial dysfunction and oxidative stress can also affect parkin function by exacerbating the occurrence of parkin mutations [114,115].

DJ-1 is an integral mitochondrial protein that may have an important role in regulating mitochondrial physiology [116], since it participates in the oxidative stress response [117,118] and protects against the loss of dopaminergic neurons [119,120]. In agreement, a previous study showed that DJ-1 knockout mice have mitochondria more vulnerable to oxidative damage [121]. It was reported that the total level of DJ-1 protein is significantly reduced in substantia nigra of sporadic cases of PD and DJ-1 complexes are reduced in cortical mitochondria of PD patients [122]. In *Drosophila*, the inhibition of DJ-1 function results in cellular accumulation of ROS, increased sensitivity to H_2_O_2_, inhibition of catalase and loss of dopaminergic neurons [123]. Furthermore, data suggests that mutations in mtDNA may also contribute to PD pathogenesis. Indeed, the level of mtDNA mutations appears to increase in pigmented neurons in the substantia nigra of human aged brain [124]. As well, the level of mtDNA deletions is significantly increased in COX-deficient neurons, thereby suggesting that mtDNA may be responsible for impaired cellular respiration [124]. Bender and co-workers [125] reported that in substantia nigra neurons from aged and PD individuals there is a high level of mtDNA deletions associated with respiratory chain deficiency contributing to neuronal loss (Figure 1). In addition, there are reports of cases where inherited mtDNA mutations lead to Parkinsonism. It was found that the Leber´s optic atrophy G11778A mutation was related to L-DOPA-responsive Parkinsonism [126]. Also the mutations in the nuclear-encoded mtDNA polymerase-γ gene have been demonstrated in patients with Parkinsonism [127]. A recent study performed in knockout “MitoPark” mice that possess a disrupted Tfam gene in dopaminergic neurons, showed that these mice have a reduced mtDNA expression and impaired respiratory chain in dopaminergic neurons and a progressive PD phenotype [128]. It was also demonstrated that dopaminergic neurons from substantia nigra possess reduced mitochondrial mass and size when compared to dopaminergic neurons from non-substantia nigra [129] supporting the idea that the selective vulnerability of dopaminergic neurons may be due to mitochondrial dysfunction in PD.

HD is an autosomal dominant neurodegenerative disorder caused by the expansion of a CAG trinucleotide repeat in the huntingtin gene [130] and is clinically characterized by chorea, psychiatric disturbances and dementia [47]. The pathogenic process in HD seems to involve transcriptional deregulation [131] and proteasome dysfunction [132]. In contrast, it was shown that the neuronal damage that occurs in a mouse model of the polyglutamine disease SCA7 can occur in the absence of ubiquitin-proteasome (UPS) dysfunction [133] and that polyubiquitylated proteins accumulate in R6/2 mice brain even with a functional UPS system [134].

Moreover, there is significant evidence of the involvement of mitochondrial dysfunction in HD [47]. Nuclear magnetic resonance spectroscopy (NMR) experiments in symptomatic HD patients revealed increased lactate levels in the cortex and basal ganglia [135]. Further, PET studies showed impaired glucose metabolism early in the disease [136,137] raising the hypothesis that glycolysis is up-regulated in order to compensate for impaired ATP production by OXPHOS. Other studies also reported decreased activities of the complexes I, II, III, and IV [138,139,140] of the respiratory chain in human HD brain in which neuronal loss was evident. In addition, in striatal neurons expressing the first 171 amino acids of huntingtin with an insertion of 82 glutamines, the overexpression of complex II subunits blocked mitochondrial dysfunction and cell death [141]. However, Guidetti and colleagues [142] reported that in presymptomatic or grade I HD brain there are no changes in the activities of complexes I-IV in the striatum. Similarly, Milakovic and Johnson [130] showed that mutant huntingtin indeed compromises mitochondrial respiration and ATP production, but these effects seem not to be related with the impairment of the respiratory chain complexes. Previous studies also demonstrated that the calcium buffering capacity is altered in lymphoblast mitochondria from HD patients as well as in brain mitochondria from transgenic mice expressing full-length mutant huntingtin [143]. Knowing that OXPHOS is dependent on mitochondrial calcium concentration [144], it could be speculated that compromised respiration in the striatal cells expressing mutant huntingtin could be due to altered mitochondrial calcium concentrations [130] (Figure 1). Another hypothesis by which mutant huntingtin could affect mitochondrial function is by altering transcription [145]. It is known that mutant huntingtin interacts with transcription factors, such as p53 [146] that is involved in mitochondrial function and oxidative stress. Bae and co-workers [147] reported that mutant huntingtin bound p53 increasing its levels and transcriptional activity leading to upregulation of BAX and PUMA, two pro-apoptotic proteins, and mitochondrial membrane depolarization (Figure 1). On the other hand, p53 supression or deletion prevented mutant huntingtin-induced mitochondrial depolarization, COX deficiency and cytotoxicity [147].

ALS is a fatal late onset neurodegenerative disorder characterized by the loss of upper and lower motor neurons leading to paralysis [24,148]. Approximately 90% of the cases are sporadic and the remaining 10% are familial [40]. However, in both cases mitochondrial and bioenergetic defects are widely implicated, being reported situations of abnormal structure, number and localization of mitochondria in ALS motor neurons and skeletal muscle [149]. Therefore, there is a strong notion that mitochondrial dysfunction may play a critical role in ALS pathology. About 20% of familial cases are caused by mutations in Cu/Zn-superoxide dismutase (SOD1) [40]. Although it was previously thought that SOD1 was exclusively a cytoplasmic protein, more recent studies demonstrated that this protein is also present in mitochondria. Evidence from transgenic mice expressing both wild-type and mutant SOD1 have shown that a fraction of cellular SOD1 is present within the intermembrane space of mitochondria [150,151] and also within the matrix affecting directly mitochondrial function and integrity [152] (Figure 1). Moreover, in SOD1-overexpressing G93A transgenic mice the morphological changes in mitochondria are the first pathological changes followed by decreased mitochondrial respiration [150,153]. Mattiazi and colleagues [154] reported that G93A transgenic mice at the onset of the disease presented compromised mitochondrial respiration and ATP synthesis that was accompanied by oxidative damage to mitochondrial lipids and proteins. In addition, decreased mitochondrial calcium loading capacity and respiratory chain complexes activity was also reported in mutant SOD1 transgenic mice [155] (Figure 1). Similarly, it was recently reported that an early functional consequence of the association of mutant SOD1 with motor neuron mitochondria is reduced capacity of the electron transport chain to limit calcium-induced depolarization [156] supporting the idea that mutant SOD1 is associated with impaired mitochondrial function. It was recently reported that in motor neurons, mutant SOD1 damages fast axonal mitochondrial transport in the anterograde direction [157]. Nevertheless, the interaction of mutant SOD1 with mitochondria is unclear. Vande Velde and co-workers [158] suggested that mutant SOD1 accumulates and aggregates in the outer mitochondrial membrane blocking protein importation to mitochondria. Others also suggest that mutant SOD1 binds to Bcl2 on the outer mitochondrial membrane blocking its antiapoptotic activity [159] (Figure 1), thereby promoting apoptosis triggered by cytochrome c release from mitochondria [160]. These evidences suggest that mitochondrial dysfunction and oxidative stress occur early and have a major role in the pathogenesis of neurodegenerative diseases.

## 4. Insulin and the Brain

For a long time it was believed that the brain was unresponsive to insulin but subsequent studies brought evidence of neuronal insulin synthesis [161,162,163], with the highest levels found in olfactory bulb, cortex, hippocampus, hypothalamus and amygdala [164]. It was also found that brain insulin and IR are independent of peripheral insulin levels [165]. However, the local synthesis of insulin remains controversial.

IR is a heterotetramer composed of two extracellular α subunits that possess a binding-site for insulin, and two transmembrane β subunits linked by disulfide bonds. Insulin binding to IR α subunits leads to insulin/IR complex internalization and autophosphorylation of the tyrosine residues of the β subunits [166,167] creating docking sites for adaptor proteins, namely insulin receptor substrate (IRS) 1 and 2, which in turn recruit and activate other proteins initiating several signaling cascades [168].

The PI3K-PKB/Akt pathway is also activated by the insulin-like growth factor (IGF-1). Insulin and IGF-1 are genetically related polypeptides that possess similar tertiary structures and considerable aminoacid identity. IGF-1 is synthesized predominantly in the liver but also in the brain and when in the circulation and tissues it is often associated to high affinity IGF binding proteins, which prolong IGF-1 half-life and modulate its interaction with the IGF-1 receptor (IGF-1R) [172]. IGF-1R is homologous to IR and trigger similar intracellular signaling events [173] such as the inhibition of GSK-3β regulating tau phosphorylation [174]. The worm insulin/IGF-1 signaling pathway is closely identical to that in mammals and is activated when an insulin-like ligand binds to daf-2, the sole worm insulin/IGF-1R, leading to a cascade of events that ends with the regulation of longevity and stress resistance [7]. Unlike worms that have only one insulin/IGF-1 signaling pathway, mammals also have the insulin/IGF-2 signaling pathway. When evaluating the effects of fat mass reduction and alterations in insulin/IGF-1 pathway in longevity using a fat-specific insulin receptor knockout (FIRKO) mice it was observed that a reduction in adipose tissue is associated with an increase in longevity probably through a reduction in insulin signaling [175]. It has been shown in mammals and worms that the decrease in IGF-1R levels leads to an increase in oxidative stress resistance and life span [176]. It has been also shown that mutations in an insulin-like signaling pathway in C. elegans influence the aggregation and toxicity of polyglutamine that is known to be intensified during aging [177]. Also, the decrease in insulin/IGF-1 signaling led to the slowing of aging together with reduced aggregation-mediated Aβ42 toxicity [178]. Freude and colleagues [179] recently demonstrated that impaired insulin/IGF-1 signaling delays Aβ accumulation and prevents premature death in Tg2576 mice, a model of AD [177]. Strategies to lengthen lifespan could be useful in the delay of the onset of aging-related diseases characterized by the appearance of misfolded and aggregation proteins.

The PI3K-PKB/Akt pathway is also activated by the insulin-like growth factor (IGF-1). Insulin and IGF-1 are genetically related polypeptides that possess similar tertiary structures and considerable aminoacid identity. IGF-1 is synthesized predominantly in the liver but also in the brain and when in the circulation and tissues it is often associated to high affinity IGF binding proteins, which prolong IGF-1 half-life and modulate its interaction with the IGF-1 receptor (IGF-1R) [172]. IGF-1R is homologous to IR and trigger similar intracellular signaling events [173] such as the inhibition of GSK-3β regulating tau phosphorylation [174]. The worm insulin/IGF-1 signaling pathway is closely identical to that in mammals and is activated when an insulin-like ligand binds to daf-2, the sole worm insulin/IGF-1R, leading to a cascade of events that ends with the regulation of longevity and stress resistance [7]. Unlike worms that have only one insulin/IGF-1 signaling pathway, mammals also have the insulin/IGF-2 signaling pathway. When evaluating the effects of fat mass reduction and alterations in insulin/IGF-1 pathway in longevity using a fat-specific insulin receptor knockout (FIRKO) mice it was observed that a reduction in adipose tissue is associated with an increase in longevity probably through a reduction in insulin signaling [175]. It has been shown in mammals and worms that the decrease in IGF-1R levels leads to an increase in oxidative stress resistance and life span [176]. It has been also shown that mutations in an insulin-like signaling pathway in C. elegans influence the aggregation and toxicity of polyglutamine that is known to be intensified during aging [177]. Also, the decrease in insulin/IGF-1 signaling led to the slowing of aging together with reduced aggregation-mediated Aβ42 toxicity [178]. Freude and colleagues [179] recently demonstrated that impaired insulin/IGF-1 signaling delays Aβ accumulation and prevents premature death in Tg2576 mice, a model of AD [177]. Strategies to lengthen lifespan could be useful in the delay of the onset of aging-related diseases characterized by the appearance of misfolded and aggregation proteins.

Another major pathway activated downstream of IR is the mitogen-activated protein kinase/extracellular signal-regulated kinase (MAPK/ERK1/2) pathway. Briefly, the cytoplasmic intermediate protein (shc) binds to IR promoting its phosphorylation. Then it binds to Grb2, which is associated with son of sevenless (SOS), a guanylnucleotide exchange factor for GTP-binding protein Ras. Binding of Grb2/SOS complex to IR activates Ras that, in turn, recruit Raf leading to MEK activation. Activated MEK phosphorylates ERK1/2 on its threonine/tyrosine residues that thereby become activated [167]. Accumulating evidence demonstrated that MAPK/ERK1/2 activity is involved in memory and learning [180,181] as well as in long term-potentiation (LTP) [182] and long term-depression (LTD) [183]. 

Both PI3K-PKB/Akt and ERK1/2 pathways are regulated by insulin and a crosstalk between them seems to exist. When PI3K-PKB/Akt is stimulated by insulin it acts antagonistically to Ras/Raf-ERK pathway and when PI3K-PKB/Akt is blocked an increase in ERK1/2 phosphorylation occurs [1]. Therefore, it is plausible to assume that insulin-mediated crosstalk between PKB/Akt and Raf is an alternative way to promote neuronal survival [1].

The specific localization of IR in the cortex and hippocampus is in agreement with evidence showing that insulin influences memory and learning [184]. Evidence from studies with rodents showed that an acute intracerebroventricular injection or an intrahippocampal administration of insulin enhances memory in a passive-avoidance task [185,186]. In addition, acute intravenous insulin enhances story recall in AD patients [187] and when given intranasally to humans, insulin is transported into hypothalamus and hippocampus without affecting blood glucose or insulin concentrations, improving effects of short-term memory functions [188,189,190]. Since peripheral glucose levels are not affected, this effect could only be due to stimulation of brain IRs [191]. Interestingly, when rats are trained on a spatial memory task, an increase in IR mRNA in the dentate gyrus and hippocampal CA1 field is observed [184]. Thereby, IR expression or function is also influenced by learning, supporting the notion that insulin contribute to normal memory function [192]. Insulin/IR have been shown to influence synaptic activities in both pre- and postsynaptic sites. At the presynaptic site insulin/IR affect catecholamine neurotransmission [193,194] and seem to be involved in neurotransmitter clearance through the regulation of synthesis and activity of dopamine, serotonine and gamma-aminobutyric (GABA) transporters [195]. Accordingly, it was recently reported that insulin has direct electrophysiological effects on central neurons that are highly influenced by GABA-inputs [196]. At the postsynaptic site, insulin/IR signaling modulates and is modulated by glutamate through N-methyl D-aspartate (NMDA) receptors activity [197,198]. In summary, insulin-signaling pathways through an intrinsic regulation, coordinates themselves to ensure synaptic plasticity, memory and learning processes and neuronal survival.

## 5. Insulin Signaling Dysregulation and Neurodegeneration

Impaired insulin/IR signaling negatively affects several functions of brain cells such as glucose homeostasis, energy metabolism and white matter fiber structure and function [199]. Neurodegenerative diseases affect a major proportion of the general population and 20% of them are associated with diabetes mellitus, increased insulin resistance and obesity, disturbed insulin sensitivity, and excessive or impaired insulin secretion [200]. Type 2 diabetes is becoming widely recognized as a risk factor for AD development and features like insulin signaling defects, Aβ accumulation and hyperphosphorylation of tau protein are possible contributors to this relation [201]. Insulin degrading-enzyme (IDE) is a metalloprotease enzyme that catalyzes the degradation of insulin following internalization of insulin and its receptor [168]. IDE also degrades soluble Aβ thereby regulating its extracellular levels by reducing aggregation and plaque formation [168]. AD brains present a reduction in IDE levels [202] and in APP mutant mice IDE overexpression reduces plaque pathology [203]. However, IDE affinity for insulin is much greater than for Aβ [204]. Accordingly, Ho and collegues [205] using an APP transgenic AD animal model demonstrated that insulin resistance caused by high fat diet is associated with a decrease in IDE levels, PI3K-Akt activity and an increase in Aβ formation. 

PKB/Akt is a player in the neuroprotection mediated by insulin signaling. In fact, data show that Akt overexpression in PC12 cells protected against Aβ induced cell death [206]. Conversely, intracellular Aβ expression inhibited both insulin-induced Akt phosphorylation and activity [207]. PKB/Akt signaling also induces the phosphorylation and inhibition of glycogen synthase kinase-3β (GSK-3β). GSK-3β is a serine/threonine protein kinase ubiquitously expressed throughout the body that possesses as substrate the protein tau [168]. In AD brains GSK-3β expression and activity is deregulated [208] and consequently tau phosphorylation is increased [209]. It has been shown that the intracerebroventricular (icv) injection of streptozotocin, an experimental model used to mimic sporadic AD, leads to defects in the insulin signaling pathways such as reduced PKB/Akt activity and increased GSK-3β activity and tau hyperphosphorylation [210,211]. In opposite, *in vitro* studies demonstrate that insulin reduce tau hyperphosphorylation by the inhibition of GSK-3β through the PI3-K pathway [212]. Also the presence of the type 4 allele of *APOE-ε4* contributes to the predisposition to AD in diabetic patients [213].

The loss of memory in early AD patients seems to involve synaptic damage caused by small Aβ oligomers, also known as Aβ-derived ligands (ADDLs) that have the ability to affect synapse composition, structure and abundance [214]. Recently, De Felice and colleagues [215] evaluated synapse pathology in mature cultures of hippocampal neurons and observed that before spine loss, ADDLs caused major downregulation of plasma membrane IRs through a mechanism sensitive to calcium calmodulin-dependent kinase II and casein kinase II inhibition. The authors also observed that the loss of IRs, and ADDL-induced oxidative stress and synaptic deterioration was prevented by insulin through IR signaling-dependent downregulation of ADDL binding sites rather than ligand competition [215]. Therefore, dysfunction of the insulin signaling may be involved in the pathological events that occur in AD brains [173]. Indeed, it has been shown that brains with advanced AD present major abnormalities in insulin and IR gene expression [216]. 

It is estimated that 50-80% of PD cases suffer from impaired glucose tolerance [217]. It has been suggested that diabetes accelerates progression of both motor and cognitive symptoms in PD [218]. PD patients also present loss of IR immunoreactivity and mRNA in the substantia nigra [219]. Indeed, previous data show that insulin production, insulin resistance and glycemic control are affected by dopaminergic drugs like bromocriptine, a D2 receptor agonist that was shown to improve insulin sensitivity in hamsters [220]. Also, dopamine transporter mRNA and activity in the substantia nigra were increased by intracerebroventricular delivery of insulin [221]. In situations of hypoinsulinemia a decrease in the amounts of mRNA dopamine transporters in the substantia nigra and dopamine concentrations in the mesolimbic cortex was observed [222,223]. Thus, a role for impaired insulin control of cellular metabolism in PD could be considered [224].

HD patients develop diabetes 7 times more often than control age-matched subjects and the decreased insulin secretion seems to be a possible explanation [225,226]. It was recently reported that besides the impairment in insulin secretion, HD patients also possess a decrease in insulin sensitivity and an increase in insulin resistance [227] suggesting that the progression of the insulin secretion defect may be a way to compensate for insulin resistance. Moreover, evidence shows that IGF-1/Akt signaling pathway could have a beneficial effect in HD since IGF-1, through the phosphorylation of huntingtin by Akt, abolished the huntingtin-mediated toxicity in striatal neurons [228]. Also, Yamamoto and colleagues [229] demonstrated that the activation of insulin receptor substrate 2 (IRS-2), a scaffolding protein that mediates the signaling cascades of insulin and IGF-1, leads to macroautophagy-mediated clearance of the accumulated huntingtin proteins. Moreover, data shows that in HD there is a dysregulation of Akt that in the latter stages of the disease is cleaved into an inactive form [230]. These observations indicate that the dysregulation of insulin/IGF-1/Akt pathway play an important role in HD progression. Altogether, these studies demonstrate that IGF-1 is a major player in HD.

ALS is also characterized by an impairment in glucose tolerance [231]. Evidence shows that insulin and/or IGF-1 promote motor neuron survival against glutamate-induced programmed cell death [232] whereas inhibitors of downstream IGF-1 signaling pathway lead to an increase in motor neuron death [232]. Accordingly, Kaspar and colleagues [233] reported that IGF-1 delay the onset of behavioral symptoms and sustains life in SOD1 mutant mice suggesting that IGF-1 signaling pathway has a key role in ALS.

In summary, alterations in the insulin and/or IGF-1 signaling pathways may contribute to the development and progression of several neurodegenerative diseases.

## 6. Role of Insulin and Insulin-Sensitizers in Neurodegeneration: Mitochondria as Potential Therapeutic Targets

Insulin/IGF-1 signaling pathways is involved in the balance of physiological processes that control aging, development, growth, reproduction, metabolism and resistance to oxidative stress [234], whereas their inhibition reduces neuronal survival by promoting oxidative stress, mitochondrial dysfunction and pro-death signaling cascade activation [235]. Evidence from the literature shows that aged rats present a decrease in mitochondrial potential and ATPase activity and increased mitochondrial oxidative damage [236]. In contrast, animals treated with IGF-1 presented an improved mitochondrial function associated with increased ATP production and reduced free radical generation, oxidative damage and apoptosis [236]. Similarly, data from our laboratory demonstrated that insulin treatment attenuates diabetes-induced mitochondrial alterations by improving the OXPHOS efficiency and protecting against the increase in oxidative stress [237,238]. It was also shown that in the reperfused brain, insulin regulates cytochrome c release through PI3K/Akt activation, promoting the binding between Bax and Bcl-xl, and preventing Bax translocation to the mitochondria [239]. *In vitro* studies demonstrated that stimulation of different cell types with insulin or IGF-1 leads to Akt translocation to mitochondria and GSK-3β phosphorylation [240], supporting a direct action of insulin/IGF-1 in mitochondria.

Evidence from the literature also shows that AD-associated impairments in energy metabolism and increased oxidative stress can promote a compensatory increase in PPAR-γ expression, which suggests that neuronal viability and function in AD could be improved by the treatment with PPAR-γ agonists [89]. Indeed, this topic has been a matter under intense discussion in the last years and several studies show a positive role for PPAR-γ agonists in AD.

PPAR-γ agonists can be broadly divided in two major classes, thiazolidinediones (TZDs) and non-TZDs [241]. The TZD agonists (also known as glitazones) include the anti-diabetic drugs pioglitazone and rosiglitazone that are FDA approved and widely prescribed for type 2 diabetes treatment, and the drug troglitazone that was initially approved but latter withdrawn [24,242]. Fatty acid derivatives such as 15 deoxi- ∆^12,14^ prostaglandin J2 (15d-PGJ2) and nitrosylated unsaturated fatty acids derivatives are considered to be potential endogenous ligands of PPAR-γ [243,244,245]. Evidence shows that *in vitro* PPAR-γ agonists suppress the induction of a proinflammatory response in microglia and the consequent production of neurotoxic inflammatory mediators [246,247,248]. Furthermore, PPAR-γ agonists suppress cytokine induced neuronal iNOS expression *in vitro*, thus preventing NO-mediated cell death of neurons [249]. The existence of PPAR-γ in the neurons may suggest a role in the regulation of neuronal susceptibility to excitotoxic damage since PPAR-γ activation by ciglitazone and by 15d-PGJ2 significantly reduced neuronal death in response to glutamate and NMDA-mediated toxicity [250]. There is also evidence that PPARs modulate mitochondrial function [251]. Fuenzalida and co-workers [252] reported that rosiglitazone treatment in neuronal cells up-regulates Bcl-2 thereby stabilizing mitochondrial potential and protecting against apoptosis. Similar results were obtained by Wu and colleagues [253] that demonstrated that rosiglitazone protected cells against oxygen-glucose deprivation (OGD)-induced cytotoxicity and apoptosis by suppressing H_2_O_2_ production, maintaining mitochondrial membrane potential, attenuating cytochrome c release and inhibiting activation of caspases 3 and 9. Moreover, OGD caused a significant suppression of Bcl-2 and Bcl-xl proteins levels that were restored by rosiglitazone pre-treatment [253]. Pioglitazone, another PPAR-γ agonist, induced mitochondrial biogenesis and reduced mitochondrial oxidative stress in a neuron-like cell line [254].

TZDs have been proposed as potential neuroprotective therapeutic agents for AD due to its effects in regulating insulin sensitivity, Aβ homeostasis, energy metabolism, inflammation and lipid metabolism [255,256,257]. The treatment of 12-month-old Tg2576 mice with pioglitazone decreased the soluble forms of Aβ but did not have any effect in Aβ plaque burden or inflammatory markers [258]. The authors suggested that those effects were due to the poor penetration of pioglitazone in the brain. However, Heneka and colleagues [255] reported that mice treated with a higher dosage of pioglitazone presented a significant decrease in microglia and astrocytes reaction, Aβ plaque load and reduced β-site of APP cleaving enzyme (BACE1) transcription and expression. Similar results were obtained by Sastre and co-workers [259], which suggest that PPAR-γ agonists can affect Aβ homeostasis. Recent data also demonstrated that rosiglitazone potentiates the ability of insulin to protect synapses against ADDLs-induced IR loss [215]. *In vitro* studies show that PPAR-γ activation protects rat hippocampal neurons against Aβ toxicity [260,261], induces up-regulation of Bcl-2 pathway, protects mitochondrial function and prevents neuronal degeneration induced by Aβ exposure and oxidative stress [252]. Indeed, rosiglitazone beneficial effects in memory and cognition seem to be mediated by the improvement of mitochondrial function [25,242], since it leads to an increase in mitochondria number and metabolic efficiency [262]. Therefore, brain mitochondrial biogenesis induced by rosiglitazone [263] is possibly due to PGC-1α, a PPAR-γ co-activator, since these co-activators regulate mitochondrial function and metabolism [264]. Recently, Qin and colleagues [265] examined human postmortem brain samples from AD and age-matched subjects and found that PGC-1α expression is negatively correlated with AD-type neuritic plaque pathology and Aβ42 contents. 

A small clinical trial involving 30 patients with mild AD or MCI revealed that 6 months of rosiglitazone treatment improve memory and selective attention [266]. A larger clinical trial involving 500 patients with mild to moderate AD revealed that rosiglitazone treatment resulted in a significant improvement in cognition in patients without ε4 allele of the apolipoprotein E (*APOE*-ε4) gene whereas patients with the *APOE*-ε4 showed no alterations in the cognitive tests [267].

Epidemiological studies show that 7% of PD patients have type 2 diabetes or suffer from insulin desensitization [268]. It was reported a significant decrease in IR in the substantia nigra [219,269] and reduced insulin-mediated glucose uptake in PD patients [270]. The fact that pioglitazone is used to treat type 2 diabetes by regulating insulin sensitivity, may suggest that some of the protective effects of this drug in PD may be due to its ability to regulate insulin signaling, glucose metabolism or lactate production [271]. The neuroprotective action of PPAR-γ agonists has been demonstrated in *in vitro* and *in vivo* studies. Pioglitazone proved to be effective in the prevention of dopaminergic cell loss in the substantia nigra pars compacta induced by MPTP-induced glial activation [272,273]. Recently, it was shown that this neuroprotection is mediated by the blockade of the conversion of MPTP to its active toxic metabolite MPP^+^ via monoamine oxidase-B inhibition (MAO-B) [274]. Pioglitazone is also able to protect dopaminergic neurons against lipopolysaccharide (LPS) mediated inflammation and consequent dopaminergic degeneration, while improving mitochondrial function and decreasing oxidative stress [275,276]. It seems that pioglitazone modulates NF-κB and Jun N-terminal kinase (JNK) pathways, which in turn inhibits cyclooxygenase (COX-2) expression [277] and/or inhibits iNOS expression and NO production through the regulation of p38 MAPK and PI-3K/Akt pathway [278]. It was also reported that rosiglitazone protects human neuroblastoma cells against acetaldehyde, an inhibitor of mitochondrial function [279]. This protection was mediated by the induction of antioxidant enzymes and increased expression of Bcl-2 and Bax [279]. Recently, the same authors demonstrated that rosiglitazone protects SH-SY5Y cells against MPP^+^-induced cytotoxicity by preventing mitochondrial dysfunction and oxidative stress [280]. These results suggest that PPARs agonists in addition to its anti-inflammatory properties also provide neuroprotection by regulating mitochondrial antioxidant enzymes expression and maintaining the balance between pro-apoptotic and anti-apoptotic gene expression. Moreover, PPAR-γ agonists are known to regulate the expression of the uncoupling proteins (UCP) [275], mitochondrial proteins that attenuate mitochondrial ROS production and limit ROS*-*induced cellular damage.

The high prevalence of diabetes in HD patients was first reported in the 70´s [281] and was soon confirmed with further studies [225,226]. Studies performed with R6/2 transgenic mice, a model of HD, revealed low insulin gene expression in the pancreas of these animals [282] that become diabetic at 12 weeks of age [283]. Recently, Quintanilla and colleagues [284] reported that mutant huntingtin-expressing cells possess significant defects in the PPAR-γ signaling pathway in comparison with cells expressing wild-type huntingtin protein. The authors also observed that rosiglitazone pre-treatment prevented the loss of mitochondrial potential, mitochondrial calcium deregulation and oxidative stress [284]. PGC-1α, being an essential transcriptional co-regulator, is an important mediator in protecting neurons against oxidative damage [285]. Evidence shows that PGC-1α is a strong suppressor of ROS production and induces the expression of ROS scavenging enzymes [286]. Moreover, it has been reported that mutant htt can affect mitochondrial function through the inhibition of PGC-1α expression [131,287]. Importantly, two independent epidemiological studies were recently published reporting that the PGC-1α gene appears to have modifying effects on the HD pathogenic process [288,289]. It has also been shown that resveratrol, an activator of sirtuin Sir2 homolog 1 (SIRT1), modulates the SIRT1-PGC-1α pathway having a neuroprotective effect against mutant huntingtin-induced metabolic dysfunction [290] supporting the idea that PGC-1α has an important role in HD. Activated SIRT1 leads to PGC-1α deacetylation resulting in its activation and consequent repression of glycolysis, increase in hepatic glucose output and modulation of mitochondrial function and biogenesis [291].

Inflammation is intimately associated with the neurodegeneration observed in ALS [292]. Therefore, PPAR-γ agonists have emerged as potential therapeutic agents in this neurodegenerative disease. Studies with ALS transgenic mice models revealed that pioglitazone treatment extended the survival of these mice by preventing a decrease in body weight and the loss of spinal cord motor neurons when compared to non-treated mice [293,294]. To what concerns the mitochondrial effects of PPARs agonists in ALS there is no data available in the literature but similarly to PD and HD there is the assumption that PGC-1α has a promising role in ALS [148]. There is evidence showing impaired or altered expression of genes in ALS that could be included in the PGC-1α target genes category [294,295]. Therefore, PGC-1α impairment could contribute to mitochondrial dysfunction in this disease [148]. In summary, evidence shows that insulin and insulin-sensitizing agents can be useful in the treatment of neurodegenerative diseases, mitochondria being one of the key targets.

## 7. Conclusions

All around the world, especially in Western societies, diabetic cases are increasing every day. Hand in hand with diabetes is the increase in longevity and age-related neurodegenerative diseases. Insulin signaling proceeds through two major pathways, PI3K/Akt and MAPK/ERK1/2 that coordinate to ensure neuronal survival and memory and learning processes. In addition to other alterations, the impairment of insulin signaling negatively impacts mitochondrial function leading, eventually, to cell degeneration and death. 

Development of new and more efficacious therapies for neurodegenerative diseases is a challenging task. TZDs that were first described for type 2 diabetes are now viewed as a potential treatment for neurodegenerative diseases that share common features such as insulin resistance, inflammation, mitochondrial dysfunction and oxidative stress. The recognition that PPAR-γ agonists have relevant neuroprotective actions is recent but yet very promising.
